# Hexagonal core-shell and alloy Au/Ag nanodisks on ZnO nanorods and their optical enhancement effect

**DOI:** 10.1186/1556-276X-9-237

**Published:** 2014-05-14

**Authors:** Junming Zhang, Boya Lai, Zuxin Chen, Sheng Chu, Guang Chu, Rufang Peng

**Affiliations:** 1 State Key Laboratory of Optoelectronic Materials and Technology, Sun Yat-sen University, Guangzhou 510275, China; 2 School of Metallurgy Engineering, Central South University, Changsha 41008, China; 3 School of Material Science and Engineering, Southwest University of Science and Technology, Mianyang615082, China

**Keywords:** Metal nanoparticle, Nanodisk, Zinc oxide, Plasmonic

## Abstract

Au and Ag hybrid hexagonal nanodisks were synthesized on ZnO nanorods' (0002) surface via a new two-step deposition-annealing method. The structural, compositional, as well as optical investigations were carried out systematically to find out the nanodisks' formation mechanism and optical enhancement effect. It was shown that the core-shell Au/Ag nanodisk can be formed under rapid annealing temperature of 500°C, while Au/Ag alloy nanodisks are formed if higher temperatures (>550°C) are applied. The optical effect from these nanodisks was studied through photoluminescence and absorption spectroscopy. It was found that the carrier-plasmon coupling together and carrier transfer between metal and ZnO contribute to the emission enhancement. Furthermore, the results suggest that the composition of nanodisk on the vicinity of metal/ZnO interface plays an important role in terms of the enhancement factors.

## Background

The interest in developing superior nanomaterials has seen tremendous progress in terms of nanofabrication, nanopatterning, and nano-self-assembly [1-3]. These progresses generated a wealth family of novel, engineered structures with desirable shape and electronic and optical properties [4-6]. These not only give researchers the foundation for basic physics phenomena that are not seen in bulk materials but also provided a wide range of application opportunities. A good example is the plasmonic nanostructures; particularly, Au and Ag nanoparticles are the most studied nanomaterials [7-9]. The mature solution-based synthesis techniques for Au and Ag nanostructures have enabled size, shape, and inter-particle spacing controllable solutions or arrays. They have demonstrated strong absorption and scattering resonance in a wide range of wavelength, which is now actively applied in functional devices and systems such as surface plasmon-enhanced Raman spectroscopy [10], solar cells [11,12], as well as lasers [13,14].

The advantages of nanomaterials are not limited to single component but should be extended to the possibilities to combine different nanocomponents into hybrid/composite structures [15,16]. Hybrid materials feature merits from two or more components and potentially synergistic properties caused by interactions between them. Interactions can be very strong as both the building blocks and separation between them have nanoscale dimensions [17,18]. For instance, it is well studied that nanoscale emitters benefit from metal nanoparticle or nanofilm surroundings [13,19,20]. In the wide bandgap semiconductor ZnO, reports have shown that by placing Au or Ag nanoparticles on ZnO nanorods or films [21,22], the ZnO's luminescence capability can be enhanced due to the carrier transfer from surface plasmon states to ZnO. More recently, we have developed a facile method to epitaxially grow Au, Ag, Pt, and Pd hexagonal/triangular nanodisks on ZnO nanorods' (0002) surface [23], in which Au and Ag nanodisks also exhibit very strong photoluminescence (PL) enhancement capability. So, metal/ZnO hybrid nanostructures are good candidate to yield high optical efficiencies in optoelectronic devices, i.e., lasers, LEDs, etc. Hence, further tuning these nanostructure's key parameters, i.e., the composition of Au and Ag inside one nanodisk, may be of substantial interest. On the other hand, since Au and Ag are with very similar lattice parameter and chemical properties, it is therefore possible to form lattice matched Ag/Au multi-layers in nanodisks by an all-solid-state synthesis process, and in this way, some desirable plasmonic structures can be achieved on ZnO nanorods' platform.

In this paper, we focus on the synthesis of Au/Ag core-shell and alloy nanodisks on ZnO nanorods' (0002) surface through a newly developed two-step deposition-annealing method, as well as the systematic characterization of their structural and optical properties. It is found that the annealing temperature determines the structural configuration of the Au/Ag composite nanodisks. Core-shell nanodisks form under the annealing temperature of 500°C, and intermixing Au/Ag alloy nanodisks start to form at the annealing temperature of 550°C. The hybrid structure's PL properties were further studied and analyzed in detail.

## Methods

The morphology and crystal structures of samples were characterized using field emission scanning electron microscope (SEM) (Carl Zeiss Leo SUPRA 55 system, Oberkochen, Germany) and transmission electron microscope (TEM) (FEI Tecnai G2 F30, E.A. Fischione Instruments, Inc., Export, PA, USA) with electron dispersive spectroscopy (EDS) mapping capability. PL measurements were carried out to characterize the optical properties of ZnO using a 325-nm He-Cd laser with an excitation power of 5 mW. An Oriel Cornerstone 260 1/4 m monochromator and a photomultiplier (Newport Corporation, Irvine, CA, USA) were used in the measurement. The absorption measurement was done by a Lambda 950 UV/VIS/NIR spectrometer (PerkinElmer, Waltham, MA, USA).

### Sample preparation

**Figure 1:**
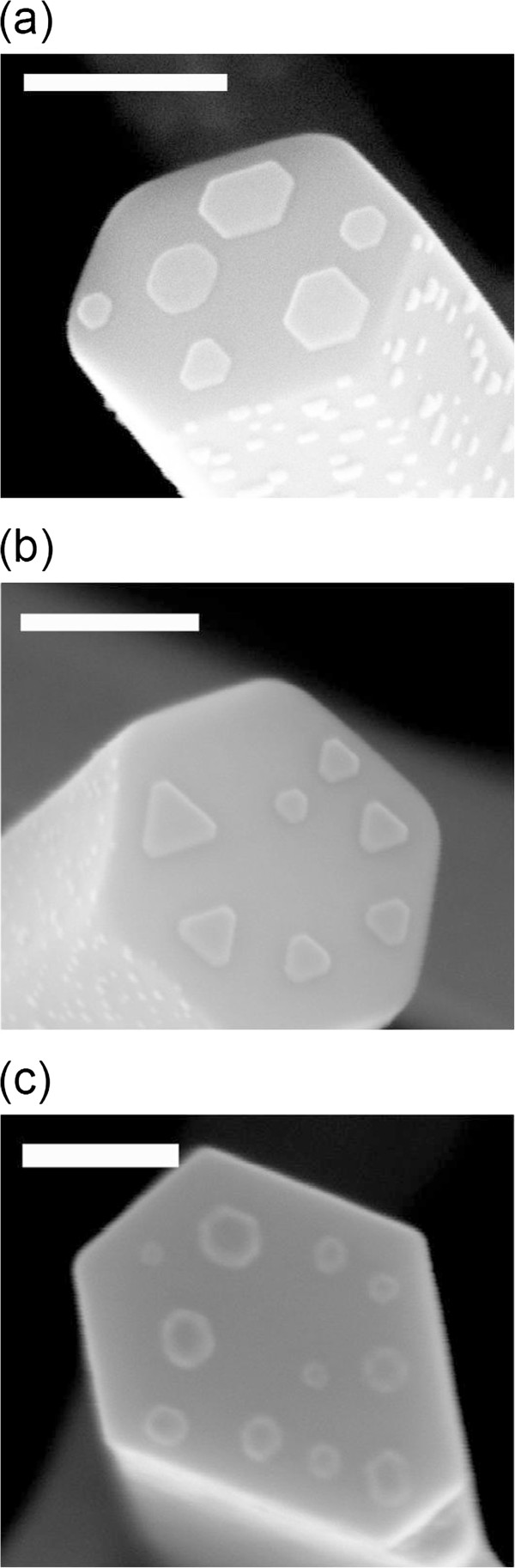
**SEM images of samples A, B, and C. (a)** Sample A: Au/Ag nanodisk annealed at 500°C, **(b)** sample B: Au/Ag nanodisk annealed at 550°C, and **(c)** sample C: Au/Ag nanodisk annealed at 600°C. Scale bar = 100 nm.

In our previous report [21], we introduced a method to epitaxially grow different elemental triangular and hexagonal metal (Au, Ag, Pt, Pd) nanodisks on ZnO nanorods' end surface. The formation mechanism of those well-defined nanodisks is attributed to the matched epitaxial relationship between metal (111) plane and ZnO (0002) plane. In the hybrid nanodisk study here, similarly, high-quality ZnO nanorods with flat (0002) surface were grown by chemical vapor deposition (CVD) method on a c-sapphire substrate and were used as the platform for the growth of metal nanodisks. The formed ZnO nanorods are with length of 1 ~ 3 μm and diameter of 100 ~ 400 nm, and for absorption measurement, aligned ZnO nanorod sample should be used. From the previous experience, the formation of single-element nanodisk is fairly reproducible and controllable; thus, the design of hybrid nanodisks is viable in a two-step strategy: to deposit and anneal Au and Ag separately on top of the ZnO (0002) surface and then anneal them to form different structures. In the experiment, 1-nm (this thickness is given by the quartz crystal of the evaporator, not the real ‘film thickness’) Au was firstly deposited by e-beam evaporation and subsequently annealed at 700°C for 60 s to enable the formation of a first layer of shape well-defined Au nanodisks. In general, as summarized in previous report [23], the growth mechanism of such hexagonal nanodisks can be briefly described: Au undergoes Volmer-Weber (VW) mode growth on ZnO. The formation process is therefore dominated by minimizing the total energy, which is dominated by the interface strain. For relatively small strain <20%, elements such as Au (111) plane will match on sixfold ZnO (0002) plane and form hexagonal nanodisks. In later experiment, this Au nanodisks layer acted as the scaffold for Au/Ag core-shell and intermixing alloy nanodisks.The sample was then put into e-beam evaporation again for 1-nm Ag capping. Since the rapid annealing is very important for the hexagonal metal nanodisks' growth, hence here we also focus on studying the annealing temperatures' effect on Ag/Au hybrid structures. Annealing was then performed on the Ag on Au/ZnO samples under different temperatures (sample A: 500°C, sample B: 550°C, and sample C: 600°C). Figure 1a,b,c shows the SEM images for samples A, B, and C, respectively. It is clearly shown that samples A and B preserve the well-defined hexagonal/triangular shapes of those single elemental nanodisks. It is found that sample C lost a noticeable degree of those defined shapes and exhibits round-shaped corners due to possible severe diffusion of Au and Ag.

**Figure 2:**
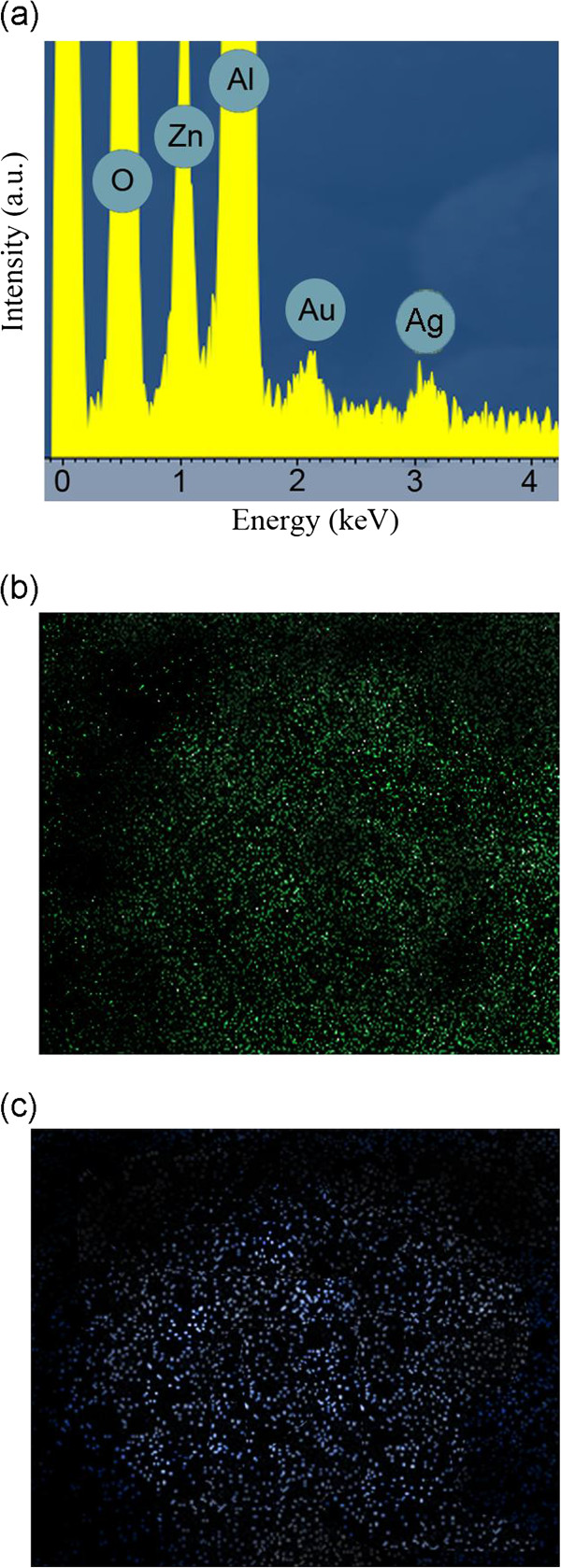
**EDS spectrum of sample A and EDS mapping for Au and Ag elements. (a)** EDS spectrum of sample A. **(b)** EDS mapping for Au element: the region of mapping corresponds to (a). Acquisition time 80 s. **(c)** EDS mapping for Ag element. Acquisition time 80 s.

Two possible cases may happen and should be clarified in the formation of these hybrid nanodisks: (1) Ag resides on top of the surface of Au nanodisks; (2) Ag forms independent hexagonal nanodisks. Since Au and Ag's lattice constants (*a*) are 4.08 and 4.09 Å, the lattice mismatch of Ag on Au is (*a*_Ag_ − *a*_Au_)/*a*_Au_ = 0.25%. Therefore, Ag residing on Au lattice will have a significantly smaller strain. However, it is still important to clarify the material distribution of Ag. X-ray EDS spectra for sample A was performed and shown in Figure 2a. It clearly resolves the signal from Au_M_ and Ag_L_. Figure 2b,c may provide insight into the elemental distribution of Ag/Au. It can be observed that both Au and Ag signals are observed on top of the same ZnO nanorod. However, whether the Au and Ag signals are from the same locations (nanodisks) is unknown due to limited resolution of EDS. In order to clarify the microstructure and Au/Ag elemental distribution, high-resolution scanning TEM with EDS mapping capability was employed for characterization.

## Results and discussion

**Figure 3:**
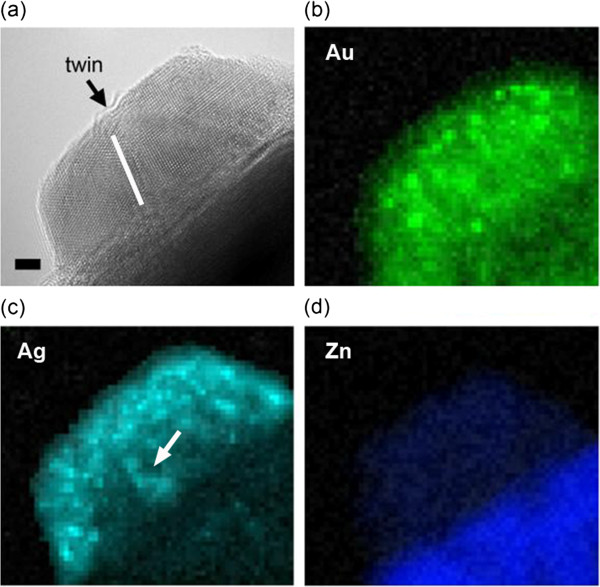
**TEM image of sample A and EDS mapping for Au, Ag, and Zn elements. (a)** TEM image of one nanodisk in sample A (low temperature annealing). Black arrow and white line indicate the twin boundary. Scale bar = 2 nm. EDS mapping for **(b)** Au, **(c)** Ag, and **(d)** Zn elements.

**Figure 4:**
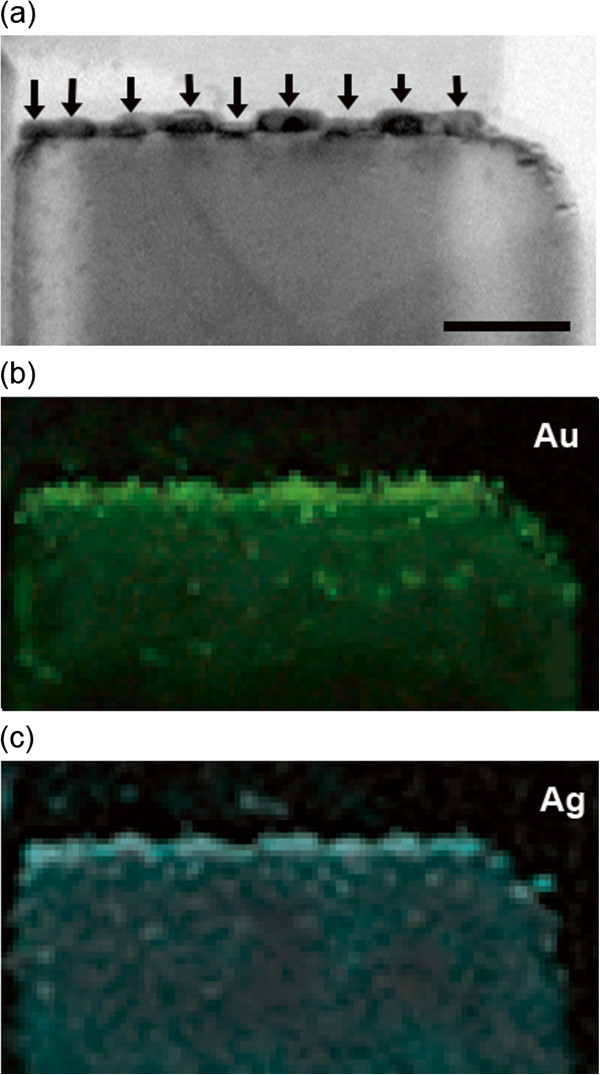
**TEM image of Ag/Au nanodisks and EDS mapping for Au and Ag elements. (a)** TEM image of Ag/Au nanodisks annealed at 500°C on a ZnO nanorod. Scale bar = 20 nm. EDS mapping for **(b)** Au and **(c)** Ag elements.

Figure 3a shows the scanning transmission electron microscopy (STEM) image of sample A, and Figure 3b,d shows the corresponding EDS mapping for elemental signal Au_M_, Ag_L_, and Zn_K_, respectively. It could be shown that the resolution of 0.5 nm is enough to locate the elements. Evidently, the concentration of Ag is higher at the outer ‘shell,,’ whereas Au concentrated at the inner regions. This is a clear indication of quasi core-shell structural Au/Ag nanodisk formation. In addition, the lattice spacing of 0.234 nm is determined from TEM, which is close to Ag and Au's (111) inter-plane distance. The (111) twin plane is observed with 72° tilted angle. This twin planes have been widely found in the previous Au nanodisks [24]. Twinning is the typical result of coalescence of multiple nanocrystals that is driven by thermal energy. Furthermore, it is also noticeable that in Figure 3b, Ag element distributes with higher density along the boundary of the twinning crystals. This is reasonable because the diffusion of Ag in Au tends to follow the defect lines in Au crystals [25]. Nevertheless, the contrast between Au and Ag is fairly clear in the EDS mapping, suggesting the quasi core-shell formation. Since Au and Ag have very similar lattice constant, the growth of Ag shell on Au nanodisks has neglectable strain; thus, in this way, the Ag/Au heterostructural nanodisk can reasonably minimize the interface energy. Interestingly, due to the small Ag/Au mismatch, it is observed that no singular Ag nanodisks actually formed on ZnO's (0002) surface, and Ag atoms all lay on Au nanodisks to minimize the interface energy. Figure 4a shows low-magnification TEM image of Ag/Au nanodisks on ZnO. Nine nanodisks were identified and marked with black arrow. In the following Au and Ag elemental mapping (Figure 4b,c), it is observed that both Au and Ag disperse in or on these nine nanodisks, suggesting that no singular Ag nanodisks were formed.

**Figure 5:**
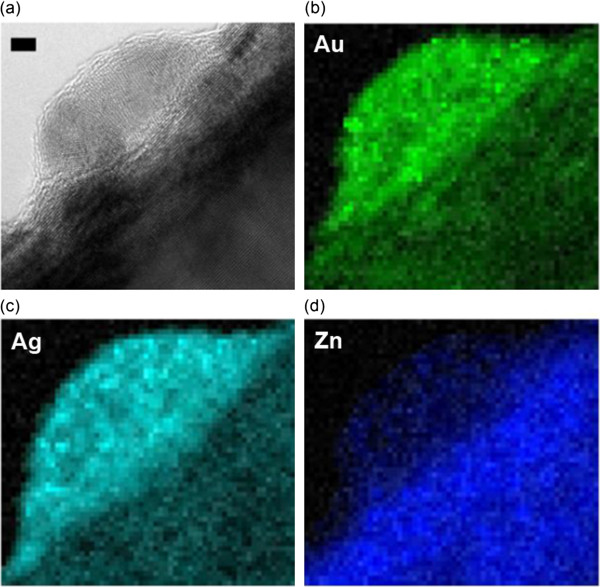
**TEM image of sample C and EDS mapping for Au, Ag, and Zn elements. (a)** TEM image of one nanodisk in sample C (high temperature annealing). Scale bar = 5 nm. EDS mapping for **(b)** Au, **(c)** Ag, and **(d)** Zn elements.

It is also known that with sufficient thermal energy, Au and Ag can easily intermix due to similar lattice structure and high inter-diffusion rate. In solution-synthesized nanoparticles, generally under relatively low annealing temperature (<200°C), Au/Ag core-shell nanoparticles start to convert to alloy nanoparticles [26]. In the solution process, annealing always needs hours to complete. As a contrary, the rapid annealing here only takes tens of seconds; thus, the status of Ag atoms will be dynamically determined by the thermal energy. In this case, relatively low temperature may not provide enough thermal energy for intermixing. As a result, with 500°C rapid annealing, sample A still displays a quasi ‘core-shell’ morphology. With longer duration of annealing or higher annealing temperatures, the mixing of Au and Ag will become much more obvious. Figure 5a,b,c,d shows the STEM images and EDS mapping of Au, Ag, and Zn for composite nanodisk sample C. In contrary to sample A, the EDS mapping signal results indicate that the Au and Ag signals are almost totally intermixed. The ratio of the Au_M_ and Ag_L_ intensity is approximately 1.2:1. Considering that the Cliff-Lorimer factor (*K*_AB_ for Au and Ag) of this EDS system is 1.52, this suggests that this alloy nanodisk is Au_0.51_Ag_0.49_. Sample B is an intermediate sample, and the STEM characterization yields an elemental distribution in between A and C (not shown here).

**Figure 6:**
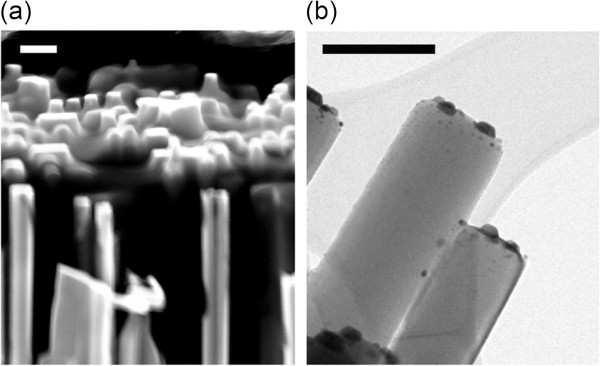
**Aligned ZnO nanorods and TEM image of Ag/Au nanodisks. (a)** Aligned ZnO nanorods with PMMA-filled inter-space. Scale bar = 100 nm. **(b)** TEM image of Ag/Au nanodisks on top of ZnO nanorods. Scale bar = 100 nm.

**Figure 7:**
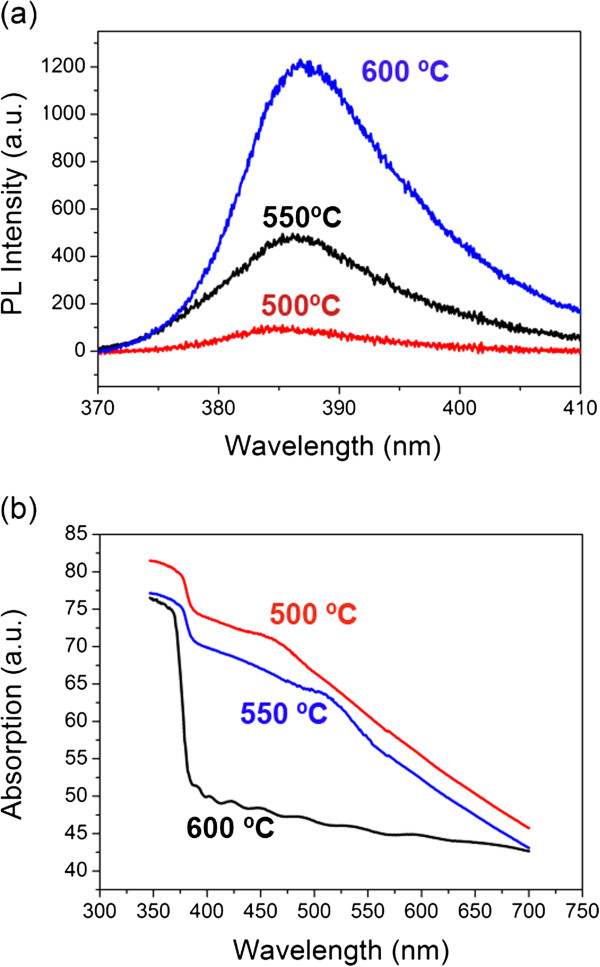
**PL and absorption spectra of samples. (a)** PL spectra under 325-nm laser excitation for samples annealed at 500°C, 550°C, and 600°C. **(b)** Absorption spectra for these samples.

Besides, the material characteristics and the optical properties of metal/semiconductors are also with profound interest. Previous studies suggest that the ability to tune ZnO's PL recombination by Au and Ag nanoparticles depends on the efficiency of carrier and plasmon coupling as well as carrier transfer between metal and ZnO [27-31]. Particularly, the authors in [31] shows that the alignment of metal energy bands with ZnO also plays an important role. Here, samples with different annealing conditions were employed to test the optical properties. The samples used in the optical characterization are aligned nanorods with relative short length to highlight metal/ZnO interface effect (approximately 1 μm), as shown in Figure 6a. In order to exclude the formation of metal nanoparticles on the side walls of ZnO nanorods, poly (methyl methacrylate) (PMMA) was spun on the sample to fill the inter-nanorod space (Figure 6a). The top surface was then rapidly cleaned by acetone and deposited with metal nanodisks. The PMMA was subsequently removed by hot acetone for the annealing process. The TEM image in Figure 6b suggests that the metal nanodots are greatly suppressed on the side walls of ZnO nanorods. This fact will enable the analysis of hexagonal nanodisks on ZnO's top surface alone rather than more randomly distributed nanodots from the e-beam evaporation process. After this treatment, the PL spectra of these Au/Ag nanodisks on ZnO nanorods are shown in Figure 7a. All samples demonstrate strong UV emissions with neglectable deep-level emissions. Evidently, 600°C annealed sample showed the strongest PL intensity, and with lower annealing temperature, PL intensity decreases evidently. The emission enhancement rate is comparable to reported metal nanostructure/ZnO systems [27-29]. The increase of ZnO near band edge emission is attributed to two possible reasons. The first reason is Purcell enhancement through carrier-plasmon coupling effect [30]. In this case, the surface plasmons of the nanodisks can couple with the ZnO photo-excited carriers (forming excitons) near the surface of the nanorods. Since the lifetime of surface plasmons is much shorter than that of electrons and holes, the carriers tend to couple with the surface plasmons of the nanodisks and then be extracted as light. As a result, the possibility of the carriers being captured by non-radiative centers will be low. Another possible reason here might be carrier transfer effect. This cannot be ruled out because there is no dielectric spacing layer between the metal and ZnO [28]. In this case, the flow of electrons from the ZnO defect level into the Au Fermi level is allowed, which increases the electron density within the nanodisk. Then, hot electrons are created in high energy states which can transfer back to the conduction band of ZnO nanorods [31]. In addition, the PL peaks redshift with higher annealing temperature, which is attributed to ZnO's rapid annealing effect (JM Zhang and S Chu, unpublished work). The authors in [32,33] investigated the Au/Ag alloy nanoparticles' plasmonic resonant characteristics and suggest that the resonant wavelength blueshifts with the increase of Ag composition, which is a result of different inter-band transitions as well as the dielectric functions of the two metals. As a result, in a nanodisk with higher Ag content, the active (resonant) wavelength will lie closer to the emission wavelength of ZnO (approximately 380 nm) and also closer to the laser excitation wavelength (325 nm). In this case, the absorption of excitation photon (325-nm laser) together with carrier/plasmon coupling is going to be stronger. Experimentally, absorption measurements were performed to examine the hybrid nanodisks' optical characteristics. The Au/Ag nanodisks were prepared on the ZnO nanorod sample and annealed in different pieces. The transmission spectra of samples annealed at 500°C, 550°C, and 600°C are shown in Figure 7b. It is observed that with higher annealing temperature, the absorption has a trend of blueshift, which is a result from plasmonic absorption band variation due to metal nanodisks. With higher annealing temperature, intermixing of Au and Ag raised higher Ag contents in the bulk of the nanodisk contacting the ZnO surface. Therefore, it caused the resonant wavelength of the alloy nanodisk blueshifts. Moreover, the work function of Au/Ag composite is reported to monotonically decrease with the increase of the Ag composition [34]. Based on a previous study [23], the work function will play a role on Ag/ZnO nanorods' PL emission: with lower work function, the band alignments favor carriers to overcome the metal/ZnO interface barrier. This factor will further assist the PL emission enhancement in annealed Au/Ag nanodisk/ZnO nanorod system.

## Conclusion

In conclusion, Au and Ag hybrid nanodisk structures were formed on the top end surface of ZnO nanorods. By varying the rapid annealing temperatures, the composite nanodisks' structure changed drastically. The core-shell and alloy Au/Ag nanodisks were achieved and characterized, while their formation mechanisms were discussed. The composite nanodisks' effect on tuning the ZnO nanorods' PL properties was further carried out. It has been found that with higher annealing temperature the PL intensity from ZnO becomes stronger, which is attributed to the shift of resonant wavelength due to composition change in the plasmonic nanodisks.

## Abbreviations

CVD: chemical vapor deposition; EDS: electron dispersive spectroscopy; PL: photoluminescence; PMMA: poly (methyl methacrylate); SEM: scanning electron microscope; STEM: scanning transmission electron microscopy; TEM: transmission electron microscope; VW: Volmer-Weber.

## Competing interests

The authors declare that they have no competing interests.

## Authors' contributions

JZ carried out the data processing and image processing and analysis, and drafted the manuscript. BL produced the sample for testing and participated in the sample test. ZC completed the sample test and helped make the sample. SC and GC conceived of the study and participated in its design and coordination, and RP helped draft the manuscript. All authors read and approved the final manuscript.
